# Fibroblast growth factor 15 overexpression and combined peroxisome proliferator–activated receptor *α* activation attenuates metabolic dysfunction–associated steatohepatitis progression in mice

**DOI:** 10.1016/j.dmd.2025.100128

**Published:** 2025-07-25

**Authors:** Hong Shi, Mary Stofan, Bo Kong, Rulaiha Taylor, Zakiyah Henry, Syeda Maliha, Daniel Rizzolo, Grace L. Guo

**Affiliations:** 1 Department of Pharmacology and Toxicology, Ernest Mario School of Pharmacy, Rutgers University, Piscataway, New Jersey; 2 Department of Infectious Disease, Third Affiliated Hospital of Sun Yat-sen University, Guangzhou, Guangdong, China; 3 Environmental and Occupational Health Sciences Institute (EOHSI), Rutgers University, Piscataway, New Jersey; 4 VA New Jersey Health Care System, Veterans Administration Medical Center, East Orange, New Jersey

**Keywords:** Bile acid, Fibroblast growth factor 15, Farnesoid X receptor, Metabolic dysfunction–associated steatohepatitis, Peroxisome proliferator–activated receptor *α*

## Abstract

Metabolic dysfunction–associated steatohepatitis (MASH) is increasing worldwide along with the obesity epidemic. However, there are limited treatments. Fibroblast growth factor (FGF)19 and its mouse ortholog FGF15, act as hormones to repress bile acid synthesis and increase energy expenditure. Activation of peroxisome proliferator–activated receptor (PPAR)*α* increases fatty acid oxidation in hepatocytes. However, a combined role of FGF19/15 with activation of PPAR*α* in the treatment of MASH is unknown. Wild-type and Fgf15 transgenic (*Fgf15* Tg) mice were fed a high-fat diet (HFD) to induce MASH, then a PPAR*α* agonist was subsequently administered to a subcohort of HFD groups to study the synergistic effect of Fgf15 overexpression and PPAR*α* activation. HFD feeding diminished glucose tolerance and increased liver fat accumulation in both wild-type and *Fgf15* Tg mice. However, *Fgf15* Tg mice demonstrated resistance to weight gain, displayed improved glucose tolerance, and attenuated liver steatosis. *Fgf15* overexpression alone reduced liver injury and the expression of genes involved in inflammation and fibrosis. The activation of PPAR*α*, both individually and in conjunction with FGF15 overexpression, led to weight loss and reduced liver steatosis. However, the combined approach with PPAR*α* activation and FGF15 overexpression resulted in an increase in liver injury and upregulation of gene expression associated with inflammation and fibrosis. In summary, Overexpression of FGF15 alone, with or without a combined activation of PPAR*α*, effectively reduces hepatic steatosis. However, the combined treatment leads to increased oxidative stress and ductular reactions, indicating a complex interplay in the pathogenesis of MASH.

**Significance Statement:**

Fibroblast growth factor (FGF)15 overexpression and peroxisome proliferator–activated receptor (PPAR)*α* activation ameliorates high-fat diet–induced hepatic steatosis. Combined PPAR*α* activation and FGF15 overexpression exacerbated high-fat diet–induced liver injury and inflammation. Overexpression of FGF15 alone or combination with PPAR*α* activation induced antioxidant response and ductular reactions.

## 1 Introduction

Metabolic dysfunction–associated steatotic liver disease (MASLD), first termed nonalcoholic fatty liver disease,^1^ is now the most common cause of chronic liver diseases globally. Metabolic dysfunction–associated steatohepatitis (MASH), previously known as nonalcoholic steatohepatitis, is characterized as ≥5% hepatic steatosis plus inflammation and hepatocyte injury with or without fibrosis. In this progressive liver condition, fat buildup triggers inflammation and cell damage, leading to fibrosis, cirrhosis, and related complications, such as hepatocellular carcinoma.^2^

MASLD occurrence has increased along with the growing worldwide obesity epidemic.^2^ The prevalence of MASLD is estimated to be 25% globally and ∼24% in the United States, with ∼20% patients with MASLD having MASH.^3^ The mechanism for MASLD progression is complex: both 2-hit and multihit hypotheses have been proposed to explain the MASLD pathogenesis.^4^^,^^5^ In these hypotheses, the initial hepatic steatosis, insulin resistance, and microbiome changes are considered to play essential roles in MASLD pathogenesis.

Fibroblast growth factor (FGF)15, human ortholog, FGF19, is highly expressed in ileal enterocytes and induced by Farnesoid X receptor (FXR) activation.^6^ FGF15/19 acts as a hormone to repress the expression of genes in bile acid (BA) synthesis in the liver through binding to its receptor FGFR4^7^ and increases energy expenditure and metabolic rate,^8^ thus reducing body weight and diabetes in leptin-deficient mice.^9^^,^^10^ Moreover, FGF15/19 also regulates hepatic lipid levels, hepatic stellate cell (HSC) proliferation, effects of gastrectomy, and protein and glycogen synthesis.^11^, ^12^, ^13^, ^14^

Peroxisome proliferator–activated receptor (PPAR)*α* belongs to the nuclear receptor NR1C subfamily^15^ and regulates lipid and glucose metabolism, energy homeostasis, and inflammation.^16^ As a master regulator of lipid metabolism, PPAR*α* plays critical roles in fatty acid (FA) synthesis and oxidation. Furthermore, acute activation of PPAR*α* also induces FGF21, which acts as an endocrine factor to increase lipolysis in white adipose tissue (WAT).^17^

Many drug targets are currently in development to treat MASH. In 2024, the US Food and Drug Administration approved the first medication for the treatment of MASH, Rezdiffra (resmetirom).^18^ However, Rezdiffra is intended to slow the disease progression of MASH and should be used along with diet and exercise. Other ongoing clinical trials are also testing candidates with diverse mechanisms of action, including glucagon-like peptide 1 and glucagon-like peptide 1/glucagon dual receptor agonist.^19^^,^^20^ Owing to being a multifactorial disease and the complex pathophysiology, a combinational therapeutic approach may be needed to effectively treat MASH.

Because of the multiple metabolic functions of FGF15/19 and PPAR*α* in the regulation of BA homeostasis and glucose and lipid metabolism, single PPAR*α* activation or FGF15/19 has been studied in several preclinical and clinical MASLD/MASH models, with the outcome not optimistic for MASH resolution. For instance, modified FGF19 has been shown to be beneficial in mouse models of MASH and cholestasis,^21^^,^^22^ suggesting a therapeutic possibility in using this target to treat MASH in humans. However, treatment with only FGF19 seems not sufficient for MASH treatment because a clinical trial to test the effectiveness of modified FGF19 in MASH treatment did not provide promising results,^21^ suggesting that additional understanding of MASH pathogenesis and better strategies to treat MASH are needed. In this study, the effect of combined PPAR*α* activation and FGF15 overexpression on the efficacy of MASH treatment was determined in a high-fat diet (HFD)-induced MASH mouse model.

## 2 Materials and methods

### 2.1 Animals and treatment

The *Fgf15* transgenic (Tg) male mice were generated on a C57BL/6J background.^23^ Age-matched C57BL/6J mice were used as wild-type (WT) controls. At 8–10 weeks of age, male Tg and WT mice were fed either a control chow diet (CD; 10% calories from lard, 0.00136% cholesterol, 70% calories from carbohydrates; Research Diets; Cat# D12450) or HFD (60% calories from lard, 0.2796% cholesterol, 20% calories from carbohydrate; Research Diets; Cat# D12492). Mice were group housed and maintained under 12-hour light/dark cycles. Food and water were provided ad libitum with total body weights and food intake recorded weekly. After 4 months on the assigned diets, a PPAR*α* agonist WY-14643 (WY; CAS# 50892-23-4; TCI Chemicals; Cat# C1323) was administered for 6 weeks to half of the mice in HFD groups by mixing the compounds with diet (0.05% w/w). The other half of the HFD mice were on vehicle. Mice were randomly assigned to each treatment.

An oral glucose tolerance test (GTT) was performed at 4 months after HFD feeding.^24^ Mice underwent overnight fasting before GTT. Blood glucose levels were detected using blood from the tail vein with a glucometer before (0 minutes) and after a single glucose oral gavage (2 g/kg, 10 mL/kg b.wt.) at multiple time points (15, 30, 60, and 120 minutes). Area under curve of the glucose level was calculated accordingly to assess insulin sensitivity in mice.

Mice were killed after 6 months of assigned feeding. Blood, liver, and WATs were collected and snap frozen in liquid nitrogen for further analysis.^25^ The animal study was approved by the Rutgers Institutional Animal Care and Use Committee.

### 2.2 Serum and liver biochemical assay

Serum activities/levels of alanine aminotransferase (ALT), aspartate aminotransferase (AST), alkaline phosphatase (ALP), triglycerides (TGs), and total cholesterol (TC) were determined by the commercially available kits purchased from Pointe Scientific. Total bile acids (TBAs) were determined by a kit from Diazyme Laboratories according to the manufacturer’s protocol.

Lipids in the liver tissue were extracted using a chloroform–methanol mixture.^26^ TG and TC in the extraction were measured using the kits from Pointe Scientific. Hepatic lipid concentration was presented as milligrams per gram of liver weight (LW).

Lipid peroxidation and oxidative stress in the liver was assessed by thiobarbituric acid reactive substances (TBARS) assay.^27^ Briefly, liver samples were homogenized in radio-immunoprecipitation assay buffer with protease inhibitors, and the level of TBARS, including malondialdehyde, was measured by the kit from Cayman Chemical (Cat# 10009055) according to manufacturer’s protocol and expressed in nanomoles of malondialdehyde per gram of liver tissue.

### 2.3 Gene expression assay

Total RNA was extracted from homogenized frozen tissue in TRIzol reagent and reverse transcribed to cDNA. Relative gene expression was determined by reverse-transcription quantitative polymerase chain reaction with Sybr Green chemistry. Relative mRNA levels were normalized to those of a housekeeping gene, *β*-actin. The primers used for real-time polymerase chain reaction are listed in Supplemental Table 1.

### 2.4 Histology and immunohistochemistry

Liver samples were sectioned and stained with H&E using the standard protocol. Immunohistochemistry for hepatic expression of cytokeratin (CK)19 and desmin was done using citrate antigen retrieval, followed by overnight incubation in 50% goat serum with rabbit anti-CK19 antibody (Abcam 52625), or goat anti-desmin (Thermofisher PA5-19063), followed by staining with the VECTASTAIN Elite ABC-HRP Kit according to the manufacturer’s instructions. Images were captured with a VS120 Slide Scanner (Olympus). Photomicrographs were taken using the VS120-S5 System (Olympus). Three random areas (400×) from each immunohistochemical specimen were selected, the images acquired, and the cells with positive staining per microscopic field quantified. For CK19 staining, each separate staining area was counted as 1 bile duct.

### 2.5 Statistical analysis

Data are represented as mean ± SD (*n* = 4–18/group). Comparison of groups was performed using a 2-way ANOVA followed by Tukey post hoc test. A value of *P* < .05 was considered statistically significant. In figures, hash represents significant difference between diets; asterisk, significant difference between genotypes; and ampersand, significant difference between PPAR*α* treatments. The statistical analysis was performed using GraphPad Prism.

## 3 Results

### 3.1 Overexpression of FGF15 ameliorates HFD-induced liver injury

**Fig. 1:**
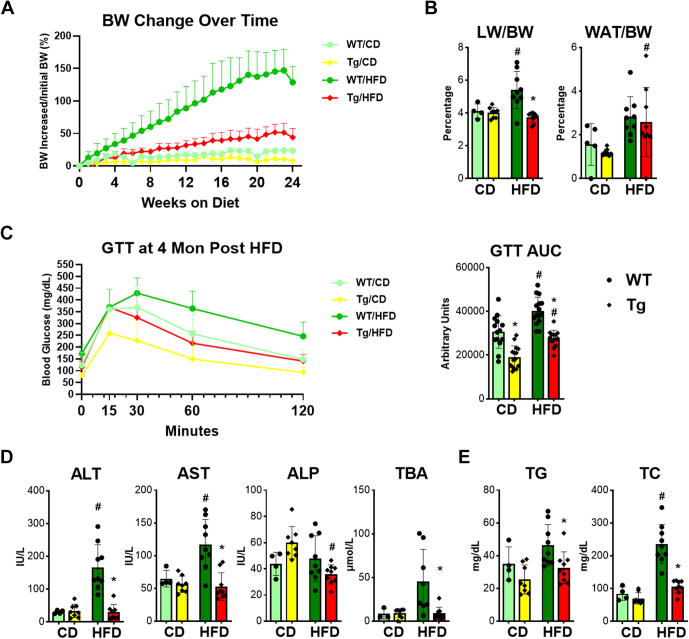
Effects of overexpression of *Fgf15* on HFD-induced change of body weight, body composition, and serum parameters. (A) Change in body weight percentage over 24 weeks. (B) Change in the liver and adipose to body weight ratios. (C) Oral GTT performed at 4 months. (D) Serum biochemical parameters showing liver injury, including activities of ALT, AST, and ALP, as well as concentration of TBA, and (E) serum TG and TC level after 24 weeks HFD feeding. Data are represented as mean ± SD. #Significant difference between diets. ∗Significant difference between genotypes.

Both WT and *Fgf 15* Tg mice steadily gained weight over the 24 weeks of feeding on HFD compared with mice on CD (Fig. 1A). Body weight change for WT mice on CD began with 0.9% ± 4.9% at week 1 and increased to 24.1% ± 1.9% at week 24, while bodyweight change of mice on HFD significantly increased from 11.0% ± 8.0% at week 1 to 150.2% ± 33.1% at week 24. However, body weight change for Tg mice on CD increased from −0.7% ± 8.3% to 10.4% ± 8.2%, while bodyweight change on HFD only increased from 1.5% ± 9.1% to 51.3% ± 14.3% after 24 weeks of feeding (Fig. 1A). LW:body weight ratio in WT mice was increased to 5.0% ± 0.9% on HFD compared with 4.1% ± 0.4% on CD. On HFD, *Fgf15* Tg mice significantly reduced LW:body weight, compared with WT mice (3.6% ± 0.6% vs 5.0 ± 0.9%) (Fig. 1B). The ratio of WAT weight to body weight (WAT:body weight) by HFD feeding increased both in WT mice (from 1.5% ± 0.4% to 2.8% ± 0.9%) and in Tg mice (from 1.2% ± 0.2% to 2.5% ± 1.3%), but no significance was found between WT and Tg mice (Fig. 1B).

A GTT test was performed at 4 months in this MASH mouse model (Fig. 1C). On both CD and HFD, Tg mice showed better glucose tolerance than WT mice; specifically, the Tg mice on either CD or HFD had a significantly smaller area under curve than WT mice on the same diet, respectively (Fig. 1C).

Six months of HFD feeding significantly increased liver injury markers, ALT and AST activities, in WT mice from 30.4 to 166.7 IU/L and 64.9 to 117.2 IU/L, respectively (Fig. 1D). Fgf15 overexpression significantly reduced HFD-induced liver injury as ALT activity dropped to 29.9 IU/L and AST activity dropped to 52.9 IU/L. Interestingly, ALP activity tended to be higher in Tg mice under CD. However, Fgf15 overexpression reduced the ALP activity on HFD, but no significant difference was found between WT and Tg mice. Serum TBA concentration elevated in WT mice after HFD feeding to 45.5 *μ*M, while Fgf15 overexpression significantly reduced TBA concentration lower than WT on both CD and HFD (Fig. 1D). Serum TG levels tended to increase on HFD, and overexpression of Fgf15 tended to decrease the TG level. Moreover, HFD feeding led to increase serum TC in both WT and *Fgf15* Tg mice (Fig. 1E), but Fgf15 overexpression attenuated serum TC levels (105.9 mg/dL) when compared with WT mice (236.0 mg/dL) (Fig. 1E).

### 3.2 Additional activation of PPARα in Fgf15 Tg mice exacerbated HFD-induced liver injury

**Fig. 2:**
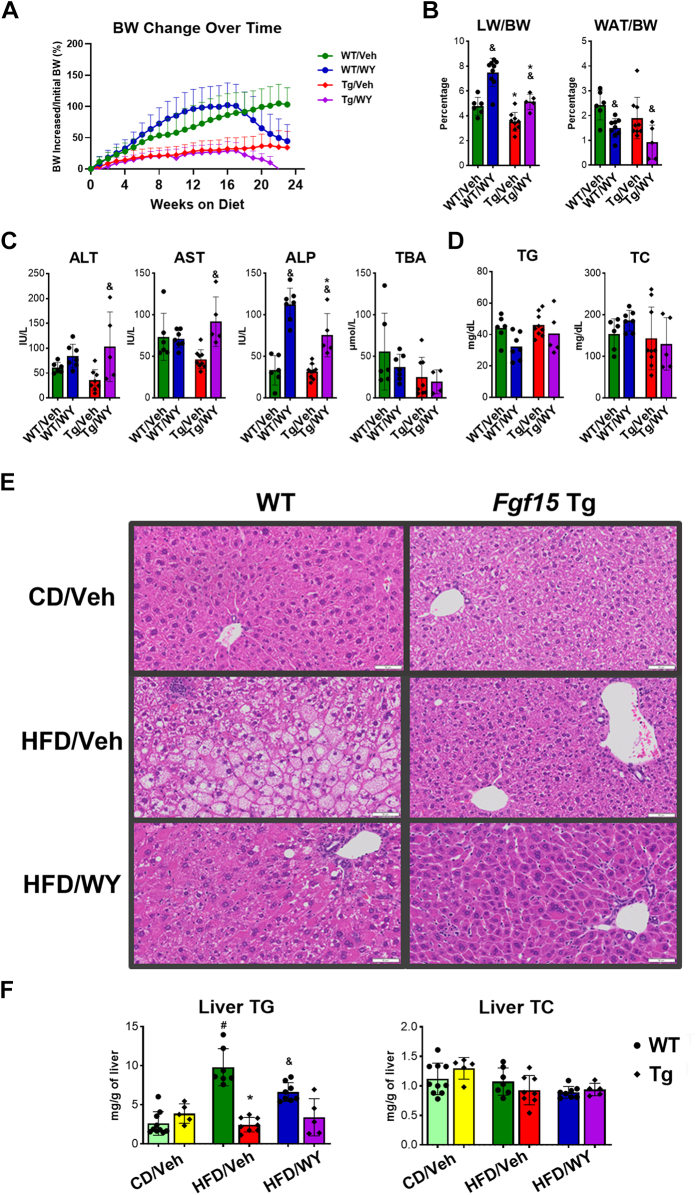
Effects of the combination of *Fgf15* overexpression and PPAR*α* activation on HFD-induced liver injury. (A) Change in body weight percentage over 24 weeks on HFD with WY treatment beginning at week 16. (B) Change in the liver and adipose to body weight ratios. (C) Serum biochemical parameters showing liver injury (ALT, AST, ALP, and TBA) and (D) serum TG and TC levels. (E) H&E-stained liver sections. Images taken at ×200 magnification. (F) Liver TG and TC levels in WT and Tg mice after HFD feeding and PPAR*α* activation. Data are represented as mean ± SD. #Significant difference between diets. ∗Significant difference between genotypes. &Significant difference between PPAR*α* treatments.

A PPAR*α* agonist WY was administered to activate PPAR*α* in mice after 4 months of HFD feeding. Body weight change on HFD for WT mice significantly increased from 8.3% ± 11.9% to 102.0% ± 35.4% but decreased to 44.5% ± 26.5% after administration of PPAR*α* agonist. In contrast, body weight change on HFD for Tg mice increased from 0.5% ± 8.0% to 35.4% ± 25.8% and dropped to 3.6% ± 17.5% after administration of PPAR*α* agonist (Fig. 2A). PPAR*α* activation significantly increased LW:body weight in both WT and Tg mice on HFD from 5.0% ± 0.9% to 7.5% ± 1.1% and 3.6% ± 0.6% to 4.9% ± 0.5%, respectively (Fig. 2B). However, PPAR*α* activation tended to decrease WAT:body weight percentage in both WT and Tg mice on HFD (Fig. 2B).

PPAR*α* activation tended to have no effect on liver injury in WT on HFD, indicated by lack of changes in serum ALT and AST levels after WY administration (Fig. 2C). However, PPAR*α* activation significantly increased serum ALT and AST levels in Tg on HFD, indicating that combined Fgf15 overexpression (Tg) and PPAR*α* activation tended to increase liver injury (Fig. 2C). Interestingly, ALP level, which indicates problems with the biliary tract and certain bone disorders, increased significantly in both WT and Tg mice after WY administration (Fig. 2C), but there was no significant difference between them. Moreover, PPAR*α* activation tended to slightly decrease serum TBA levels without significance in both WT and Tg mice (Fig. 2C). Serum TG and TC levels remained unchanged by Fgf15 overexpression alone or in combination with PPAR*α* activation (Fig. 2D).

There was no significant difference observed in histology between WT and Tg mice on the CD diet (Fig. 2E). Six months of HFD feeding induced severe steatosis and inflammation in WT mice, characteristic signs of MASH. However, Tg mice showed no signs of steatosis and only minimal inflammation under HFD (Fig. 2E). PPAR*α* activation significantly reduced steatosis in both WT and Tg mice, and only enlarged hepatocytes and some residual steatosis around the portal triad area were observed after WY administration. The lipid contents in the liver were extracted, and total hepatic TG and TC were determined (Fig. 2F). HFD feeding increased the TG accumulation, while PPAR*α* activation reduced the TG content in WT mice. However, TG level in Tg mice showed no significant change under HFD feeding or PPAR*α* activation (Fig. 2F). In addition, hepatic TC level showed no significant change between WT and Tg mice either under HFD feeding or PPAR*α* activation (Fig. 2F).

### 3.3 Overexpression of Fgf15 alone or in combination with PPARα activation disrupted the expression of genes in hepatic BA and lipid homeostasis

**Fig. 3:**
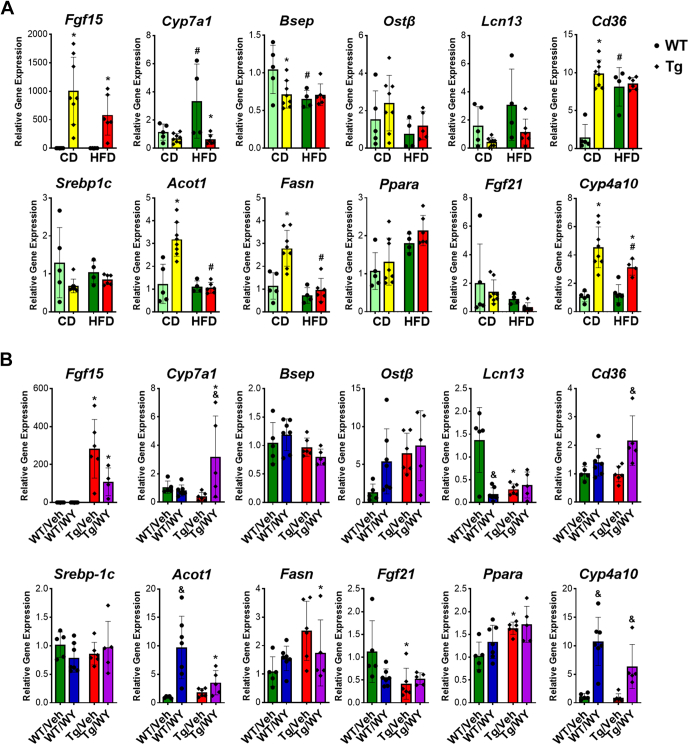
Effects on overexpression of *Fgf15* alone and in combination of PPAR*α* activation on HFD-induced gene expression changes involved in BA homeostasis and lipid homeostasis. (A) Effects of overexpression of *Fgf15* on HFD-induced gene expression change. (B) Effects of combination of *Fgf15* overexpression and PPAR*α* activation on HFD-induced gene expression change. Data are represented as mean ± SD. #Significant difference between diets. ∗Significant difference between genotypes. &Significant difference between PPAR*α* treatments.

We first determined the effects with HFD only in the WT and Tg mice. The overexpression of FGF15 was confirmed in Tg mice through measurement of hepatic and ileal expression of Fgf15 (Fig. 3A) and was further confirmed by the gene expression of cholesterol 7*α*-hydroxylase (Cyp7a1), encoding the rate-limited enzyme involved in classical pathway of BA synthesis. As expected, Fgf15 overexpression strongly suppressed *Cyp7a1* gene expression (Fig. 3A). The mRNA levels of bile salt export pump (Bsep), which is crucial for the transport of bile salts from the liver into the bile showed a decrease in WT mice with HFD feeding, but not in Tg mice. The mRNA levels of organic solute transporter (Ost)*β* that can be induced by cholestasis in the liver, exhibited a slight decrease but no significant difference between WT and Tg mice with HFD. The mRNA levels of lipocalin (Lcn)13 that has been shown to be positively associated with basal FXR activities in mice from our previous study^24^ increased in both WT and Tg mice on HFD, but clearly with levels much lower in Tg mice (Fig. 3A).

Hepatic fat accumulation results from an imbalance between lipid acquisition and lipid disposal, consisting of 4 major processes: uptake of circulating FAs, de novo lipogenesis, FA oxidation, and export of TG in the form of very-low-density lipoprotein. The mRNA levels of CD36, a membrane protein that facilitates long chain FA uptake, were induced by Fgf15 overexpression and HFD feeding. However, there was no significant difference between WT and Tg mice after HFD feeding (Fig. 3A).

HFD reduced de novo lipogenesis in both WT and Tg mice compared with CD diet, shown by reduction in the gene expression of sterol regulatory element-binding protein 1c, a transcription factor primarily controls the synthesis of FAs, acyl-coA thioesterse 1, a cytosolic enzyme involved in FA metabolism, and fatty acid synthase enzyme that carries out FA synthesis (Fig. 3A). The Tg mice exhibited significantly higher expression levels of lipogenesis under CD, but there was no significant difference between WT and Tg mice after HFD feeding.

We then determined the combined effects of PPAR*α* activation and Tg on the expression of genes critical for MASH development. Activation of PPAR*αα* increases FA oxidation, thermogenesis, and weight loss. Under CD, there was no significant difference in the gene expression of PPAR*α* between WT and Tg mice. However, both WT and Tg mice exhibited increased expression of PPAR*α* after HFD feeding, with Tg mice showing a more pronounced increase (Fig. 3A). FGF21 can be induced by acute PPAR*α* activation and acts as a hormone to mobilize adipose tissue lipolysis. The hepatic mRNA levels of Fgf21 decreased in both WT and Tg mice after HFD and were not induced by WY treatment. We further measured FGF21 protein levels in the serum by ELISA method, and the result confirmed no induction of FGF21 protein (data not shown). The cytochrome P450 4A10 gene, a well-recognized as a target gene for PPAR*α* activation, was induced in both WT and Tg mice with WY treatment (Fig. 3A).

We then checked the expression of genes in BA homeostasis. With no effect on Fgf15 mRNA levels in WT mice, PPAR*α* activation slightly reduced hepatic Fgf15 mRNA levels in the Tg mice (Fig. 3B). Interestingly, Cyp7a1 mRNA levels were markedly suppressed by Fgf15 overexpression but increased when in combination with PPAR*α* activation. In contrast, WY treatment did not alter mRNA levels of Fgf15 in WT mice. Moreover, PPAR activation slightly increased the mRNA levels of Bsep in WT mice but slightly decreased them in Tg mice, resulting in a relatively large reduction of Bsep with the WY treatment in the Tg mice compared with WT mice. Both Fgf15 overexpression and PPAR*α* activation tended to increase Ost*β* mRNA levels, which was not further enhanced by the combined treatment. Interestingly, although both Ost*β* and Lcn13 are direct FXR target genes in the liver, the mRNA levels of Lcn13 showed exactly opposite expression in comparison with Ost*β*. However, the combinational treatment did not further enhance the effect (Fig. 3B).

PPAR*α* activation increased *Cd36* gene expression in both WT and Tg mice with more induction in Tg mice, indicating more FA uptake in the liver. However, PPAR*α* activation had no effect on *Srebp-1c* gene expression but increased *Acot1* and *Fasn* gene expression in WT mice. These same genes were not changed in Tg mice, indicating that PPAR*α* activation increased FA synthesis in WT but not Tg mice (Fig. 3B). PPAR*α* activation did not change *Pparα* gene expression itself but significantly increased its target gene cytochrome P450 4A10 expression in both WT and Tg mice (Fig. 3B), reflecting the activation of the PPAR*α* pathway and significant induction of lipid oxidation. Interestingly, chronic PPAR*α* activation did not induce another PPAR*α* target *Fgf21* gene expression (Fig. 3B).

### 3.4 Overexpression of Fgf15 alone or in combination with PPARα activation contributed to inflammation and fibrosis in MASH

**Fig. 4:**
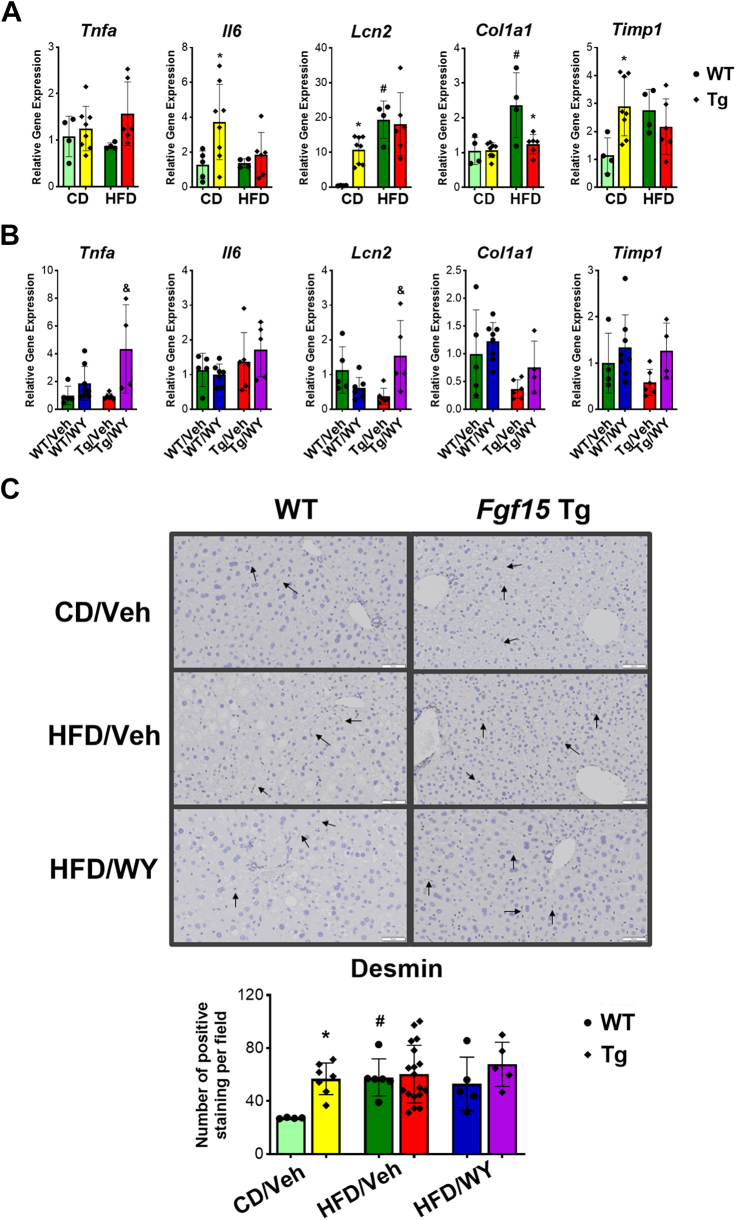
Effects on overexpression of *Fgf15* alone and in combination of PPAR*α* activation on HFD-induced gene expression changes involved in liver inflammation and fibrosis. (A) Effects of overexpression of *Fgf15* on HFD-induced gene expression change. (B) Effects of combination of *Fgf15* overexpression and PPAR*α* activation on HFD-induced gene expression change. (C) Immunohistochemistry staining and the average count of positive staining per microscopic field (×400) of desmin in HSCs. Data are represented as mean ± SD. #Significant difference between diets. ∗Significant difference between genotypes. &Significant difference between PPAR*α* treatments.

HFD did not change mRNA levels of the inflammatory cytokines, tumor necrosis factor (TNF)*α*, and interleukin (IL)-6 in WT mice but increased those of Lcn2 (Fig. 4A). *Fgf15* overexpression tended to have no effect on HFD-induced inflammation, observed by no further change of TNF*α*, IL6, and Lcn2 mRNA levels in Tg mice on HFD. *Col1a1* that encodes type I collagen and *Timp1* that encodes tissue inhibitor of metalloproteinases 1, an enzyme to disrupt the degradation of extracellular matrix, are fibrogenic genes that were both significantly induced with HFD in WT not Tg mice (Fig. 4A). WY treatment tended to increase the mRNA levels of all these genes in the Tg mice, but a synergistic effect was only seen for the inflammatory gene *Tnfα*, but not for *Il6* and *Lcn2* in WT (Fig. 4B).

Desmin expression is generally present in quiescent HSCs but exhibit higher levels in activated HSCs. Liver tissue sections were stained for desmin to view the overall presence of HSCs (Fig. 4C). Tg mice had higher level of HSCs cells, while HFD increased HSCs in both mice but without significance in Tg mice. Regardless of genotype, PPAR*α* activation tended to have no effect on the HSCs present in both mice (Fig. 4C).

### 3.5 Overexpression of Fgf15 alone or combination with PPARα activation induced antioxidant response and ductular reactions

**Fig. 5:**
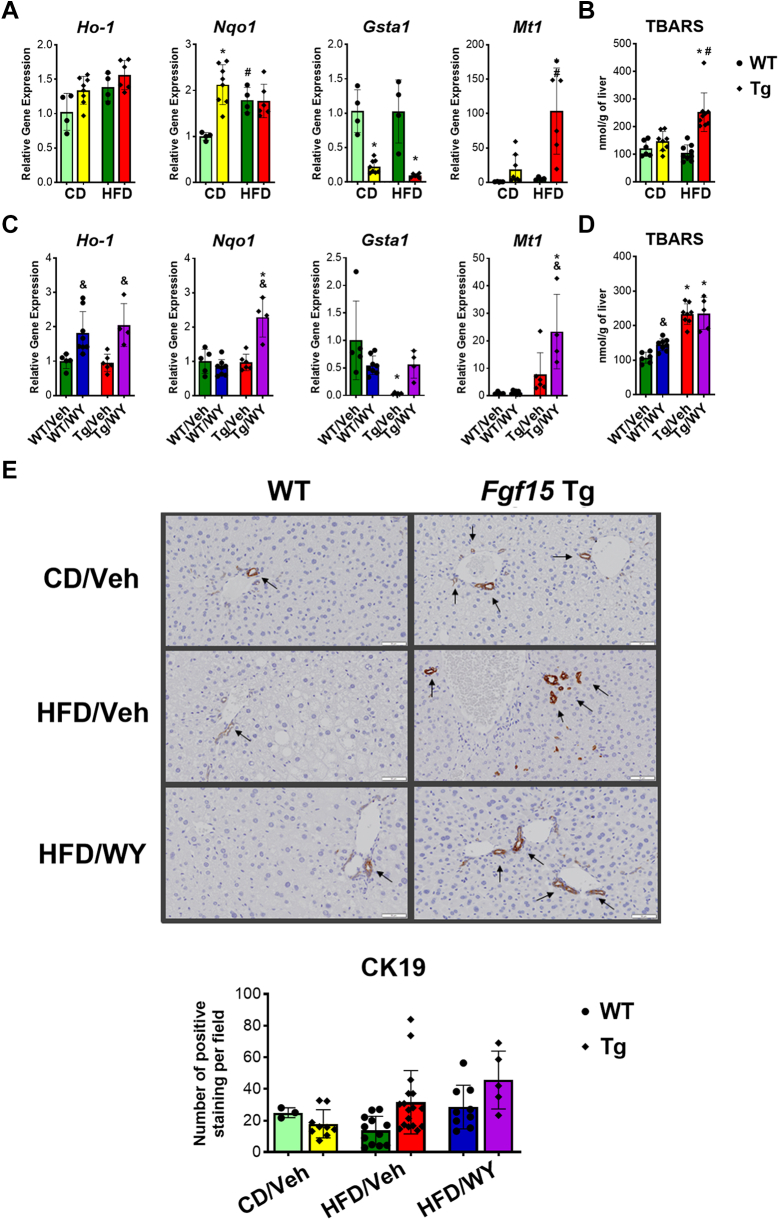
Effects on overexpression of *Fgf15* on HFD-induced (A) gene expression changes involved in antioxidant response and (B) oxidative stress in the liver. Effects on *Fgf15* overexpression in combination of PPAR*α* activation on (C) gene expression changes involved in antioxidant response and (D) oxidative stress in the liver. (E) Immunohistochemistry staining and the average count of positive staining per microscopic field (×400) of CK19 in cholangiocytes. Data are represented as mean ± SD. #Significant difference between diets. ∗Significant difference between genotypes. &Significant difference between PPAR*α* treatments.

Heme oxygenase (Ho)-1, NAD(P)H:quinone oxidoreductase (Nqo)1, glutathione S-transferase *α* (Gsta)1 and metallothionein 1A (Mt1) are essential for maintaining cellular redox balance and protect cells from damage caused by oxidative stress. The mRNA levels of Ho-1, Nqo1, and Mt1 induced significantly after HFD feeding in WT mice. Overexpression of Fgf15 tended to increase gene expression of Ho-1, Nqo1, and Mt1 on both CD and HFD, However, showed an opposite effect on *Gsta1* mRNA levels (Fig. 5A). Lipid peroxidation and oxidative stress in the liver was measured by TBARS assay, indicating HFD significantly increased the oxidation stress in Tg but not in WT mice (Fig. 5B).

PPAR*α* activation on HFD increased the mRNA levels of Ho-1 and Mt1 but showed no effects on Nqo1 and Gsta1 in WT mice. However, PPAR*α* activation significantly increased *Ho-1*, *Nqo1*, *Gsta1*, and *Mt1* gene expression in Tg mice (Fig. 5C). Moreover, PPAR*α* activation significantly increased oxidative stress in WT but did not further increase it in Tg mice (Fig. 5D).

The presence of CK19 has been used as a marker for cholangiocytes. Increased CK19 staining was observed in Tg mice than in WT mice on HFD. Moreover, HFD reduced CK19 staining in the WT mice but markedly increased those in Tg mice. WY treatment seemed increasing the CK19 staining counts in both WT and Tg mice. However, WY treatment tended to reduce the intensity of CK19 staining in the Tg mice (Fig. 5E).

## 4 Discussion

MASH is a huge medical burden globally, and there are limited treatment options to reduce the disease development or progression. MASH development is tightly associated with excessive calorie intake and BA dysregulation. BA homeostasis is critically regulated by the FXR conducted orchestration between the liver and intestine, with FGF15/19 being the most important downstream mediator induced in the gut to regulate hepatic and systemic crosstalk.^28^ In this study, the potential protection of mice from MASH development by Fgf15 overexpression alone or in combination with PPAR*α* activation was investigated using an established HFD-induced MASLD mouse model.^13^^,^^24^^,^^25^ This model reflects human MASLD progression with simple steatosis occurring at 2 months, MASH (steatosis plus inflammation) around 4 months, and MASH plus fibrosis at 6 months of HFD feeding. Histologically, WT mice on HFD exhibited MASH characteristics, including macrovesicular steatosis, infiltration of inflammatory cells, moderate fibrosis, and liver injury after 6 months of feeding. However, HFD alone in mice can induce obesity and hepatic steatosis but might not induce significant MASH progression, including liver damage, inflammation, and fibrosis. Mice generally exhibit less severe inflammation and fibrosis than human conditions.^29^ In our mouse model, we observed mild change in gene expression associated with inflammation and fibrosis as well.

Overexpression of Fgf15 can prevent obesity and MASLD development in this study. Compared with WT mice, Fgf15 Tg mice on HFD resisted weight gain, showed no signs of steatosis, and became more insulin sensitive. However, slight inflammation and partial portal triad cell proliferation (portal reaction)^28^ were accompanied with the beneficial changes. The reversal of hepatic steatosis by FGF15 might be through 2 mechanisms, FGF15 could directly promote the metabolic rate and energy expenditure on brown adipose tissue^9^ and FGF15 could also suppress BA synthesis in the liver, thus decrease the BA pool in the body and indirectly reduce intestinal lipid absorption. The activation of PPAR*α* has also been known to increase hepatic FA oxidation and reduce TG levels. Indeed, the WY treatment reduced obesity and improved hepatic steatosis.

Although the combination of Fgf15 overexpression and PPAR*α* activation had an excellent effect on promoting weight loss and reducing hepatic steatosis, the dual treatments led to increased liver injury and expression of genes in inflammation, fibrosis, and antioxidation. These results were a surprise to us because they seemed to be less additive or synergistic than expected, and different from what other studies had reported.^30^, ^31^, ^32^ It is well known that PPAR*α* activation in the liver reduces hepatic steatosis by increasing the lipid oxidation. However, emerging study also found that intestinal PPAR*α* activation promotes nonalcoholic steatohepatitis progression by inducing the expression of liver FA–binding protein 1 and thus increasing FA–binding protein 1–mediated uptake of dietary FAs,^33^ indicating the tissue-specific functions for PPAR*α* activation in lipid metabolism, which could also explain the different outcomes between this and previous studies.

The regulation of hepatic lipid metabolism by PPAR*α* is believed well conserved,^34^ and animal experiments also indicated the potential benefits of PPAR*α* activation in improving MASLD in humans.^16^^,^^31^^,^^35^ However, evidence suggests that species differences exist between rodents and humans in the biological responses to ligand activation of PPAR*α*. PPAR*α* has a lower expression level in human liver compared with that in mouse liver.^34^ In addition, long-term activation of PPAR*α* leads to excessive cell proliferation and development of liver tumors, particularly hepatocellular carcinoma, which is primarily observed in rodents and not readily translated to humans.^36^ Moreover, clinical studies showing that patients treated with synthetic PPAR*α* activators, bezafibrate and fenofibrate, showed only mild improvements,^37^, ^38^, ^39^ suggesting that long term species-effects of PPAR*α* agonists need to be further investigated in order to know the limitation of this model in respect of PPAR*α* activation.

Our study also showed that a chronic activation of PPAR*α* improves steatosis independent of FGF21 induction. FGF21 is also an endocrine FGF induced in both mice and humans in response to fasting and after acute activation of PPAR*α*.^17^^,^^40^ PPAR*α* was shown to be a key regulator of FGF21 and PPAR response elements were identified in both *FGF21/Fgf21* promoters.^41^ However, neither Fgf21 mRNA in the liver nor serum FGF21 protein was induced by chronic PPAR*α* activation in our mice. The lack of Fgf21 induction by chronic PPAR*α* activation could be due to the increased oxidative stress and activation of the Nrf2 signaling pathway after WY treatment. At a cellular level, oxidative stress occurs when harmful molecules like reactive oxygen species, lipid peroxides, and electrophiles overwhelm the cell’s capacity to remove them.^42^^,^^43^ Activation of the Nrf2-antioxidant response element signaling pathway is a major mechanism in the cellular defense against oxidative stress.^44^ In this study, overexpression of Fgf15 alone did not have any effects on activation of the Nrf2 signaling pathway, but WY treatment tended to increase oxidative stress in Tg mice, thus suspected NRF2 activation in our combination treatment group may have also inadvertently suppressed FGF21 in our study.

Interestingly, Fgf15 overexpression in HFD conditions tended to induce ductular reaction and bile duct proliferation. Ductular reaction occurs typically in biliary obstruction and is characterized by proliferation of bile ductules. These Tg mice had very low BA levels with overexpression of FGF15 in both hepatocyte and enterocyte^23^ and have less liver injury under HFD feeding, which did not suggest cholestasis but rather there might be a crosstalk between hepatocyte and cholangiocytes with super physiological concentration of FGF15. This area needs further investigation in the future.

In summary, to our knowledge, this is the first study to show that Fgf15 overexpression may be protective against MASH development in mice. In combination with PPAR*α* activation, better improvement was achieved for obesity, insulin resistance, and hepatic steatosis, independent of FGF21 induction. However, this marked efficacy was accompanied with increased bile duct proliferation and increased inflammation, cautioning the safety of the combination of FGF19 and PPAR*α* activation in the treatment of obesity and MASLD.

## Conflict of interest

The authors declare no conflicts of interest.
