# Supplementary material for: Fibroblast growth factor 15 overexpression and combined peroxisome proliferator–activated receptor α activation attenuates metabolic dysfunction–associated steatohepatitis progression in mice
Source: Drug Metab Dispos. 2025 Jul 25;53(9):100128. doi: 10.1016/j.dmd.2025.100128 (PMC12597547; doi:10.1016/j.dmd.2025.100128)
Supplement: Supplementary Table 1 [file mmc1.docx]

| **Supplemental Table 1. qPCR Primer Sequences** | | |
| --- | --- | --- |
| **Target** | **Forward Primer** | **Reverse Primer** |
| *β-Actin* | GCGTGACATCAAAGAGAAGC | CTCGTTGCCAATAGTGATGAC |
| *Acot1* | CGCAGCCACCCCGAGGTAAA | TCTCAGGATAGTCACAGGGGGT |
| *Bsep* | CTGCCAAGGATGCTAATGCA | CGATGGCTACCCTTTGCTTCT |
| *Cd36* | GATGACGTGGCAAAGAACAG | TCCTCGGGGTCCTGAGTTAT |
| *Col1a1* | ACTGTCCCAACCCCCAAAG | CGTATTCTTCCGGGCAGAAA |
| *Cyp4a10* | CTCCAAGTGGCCTCTGTGCT | TAAGTAGGCCTTGCTTCCCCA |
| *Cyp7a1* | AACAACCTGCCAGTACTAGATAGC | GTGTAGAGTGAAGTCCTCCTTAGC |
| *Fasn* | GCTGCGGAAACTTCAGGAAAT | AGAGACGTGTCACTCCTGGACTT |
| *Fgf15* | GCCATCAAGGACGTCAGCA | CTTCCTCCGAGTAGCGAATCAG |
| *Fgf21* | AAAGCCTCTAGGTTTCTTTGCCA | CCTCAGGATCAAAGTGAGGCG |
| *Gsta1* | CGCCACCAAATATGACCTCT | TTGCCCAATCATTTCAGTCA |
| *Ho-1* | TGAATCGAGCAGAACCAGC | ATGAACTCAGCATTCTCGGC |
| *Ibabp* | GGTCTTCCAGGAGACGTGAT | ACATTCTTTGCCAATGGTGA |
| *Il6* | CAACGATGATGCACTTGCAGA | GGTACTCCAGAAGACCAGAGG |
| *Ostβ* | GTATTTTCGTGCAGAAGATGCG | TTTCTGTTTGCCAGGATGCTC |
| *Ppara* | ACAAGGCCTCAGGGTACCA | GCCGAAAGAAGCCCTTACAG |
| *Lcn2* | AATGTCACCTCCATCCTGGTCA | CCACTTGCACATTGTAGCTCT |
| *Lcn13* | ACAATGGTACCTACCCAGTCACA | ACTCACGGCAATGACCATTGTTCC |
| *Mt1* | CAAGAACTGCAAGTGCACCTC | CGCCTTTGCAGACACAGC |
| *Nqo1* | TGAATCGAGCAGAACCAGC | ATGAACTCAGCATTCTCGGC |
| *Srebp1c* | GGAGCCATGGATTGCACATT | GCTTCCAGAGAGGAGGCCAG |
| *Timp1* | CCACCTTATACCAGCGTTAT | CTGGGACTTGTGGGCATATC |
| *Tnfα* | ATGGCCTCCCTCTCATCAGT | GCTCCTCCACTTGGTGGTTT |
